# Hospital and Institutional News

**Published:** 1917-01-20

**Authors:** 


### HOSPITAL AND INSTITUTIONAL NEWS.

### THE OWNERSHIP OF EXTRACTED BULLETS.

When bullets or other projectiles are extracted from wounded sailors or soldiers there occasionally arise differences of opinion as to the subsequent appropriation of the extracted missile. Some surgeons are keen to form a collection of these "souvenirs" of their skill, and rather begrudge handing them over to the patients from whom they have been extracted. Most of the patients, on the other hand, though not all, are very anxious to retain the extracted metal for themselves; and many are the injunctions to ward sisters in the base hospitals and elsewhere not to let the precious memento get lost before the victim recovers from his anæsthetic and can take custody of his trophy. As a general rule it is conceded that the wounded hero has the best moral title to his bullet; and if he wants it he is, in the British Army, allowed to have it; if he does not, as occasionally happens, the surgeon and the ward sister have been known to "toss up" for its possession, though the former has the stronger claim of the two, one would say. So far as we know, the British spirit of compromise and common sense has hitherto prevailed to such an extent that no dispute about these highly prized, though intrinsically almost valueless, pieces of property has yet come before a court of law. In Italy, however, where Latin logicality doubtless prevails more than in Britain, as it does also in France, a lawsuit on this subject has recently been decided in a town near the frontier and not far from the Front; and the arguments adduced are decidedly interesting.

### A CASE WORTHY OF PORTIA.

A surgeon by a difficult operation removed from an officer (according to the *People*) a big piece of a large-calibre shell. On his recovery the wounded man claimed the souvenir, but the surgeon stoutly refused to give it up. He argued that but for his skill the shell would have remained buried in the officer's body, and it had therefore become his exclusive property. The judge rejected this plea. He found that the projectile, once discharged from the gun that fired it, ceased to belong to either the soldier who fired it or to the country which entrusted it to him, and so became a "res nullius" which any finder is entitled to appropriate. Some lawyers, it is said, disagree with this judicial pronouncement; there are those who maintain that the surgeon is in the right, and there are others who say that the projectile still belongs to the nation from whose gun it was fired. If that is so, there must be a good deal of German property in France and England by this time, as well as a good deal of French and British in Germany! On the whole, the judge's findings, whether or no they be good law, seem to us to be substantially just and sensible. The name of the frontier town is not stated, but obviously legal proceedings on points such as this ought most appropriately to be heard in Venice; and possibly this may actually have been the case.

### GRIMSBY HOSPITAL RECORDS.

The year 1916 has been a record one for the Grimsby and District Hospital so far as donations are concerned. The salient features were the endowment of a "Jack Cornwell" cot and of a "Lloyd George" bed; a record contribution of £515 17s. 1d. by the working men; and a record donation of £300 from the Wellow Waits, who collected this amount by singing at Christmas-time. The establishment of the "Jack Cornwell" cot is largely due to the personal efforts of the chairman of the management committee, Alderman R. W. Roberts, as most of the £1,333 0s. 2d. raised has come from his business friends on the fish docks. Young Cornwell, V.C., died in the hospital, and this fact brought an offer from Mr. F. L. Elwell, a well-known artist of Beverley to paint a portrait of the boy. Alderman Roberts had previously launched his "Jack Cornwell" cot project, and the unveiling of the portrait by Rear-Admiral Stuart Nicholson was responsible for adding largely to the endowment fund. The endowment of the "Lloyd George" bed is due to the generosity of Mr. Alec L. Black, a local shipowner. Mr. Black offered a cheque for £1,000 for the purpose, in honour of the Premier, as an appreciation of his recent efforts. The Premier wrote giving his consent to the proposal, and said he was much touched by Mr. Black's personal references. The working men's collection of £515 17s. does not include a donation of £63 from the employees of Messrs. Charlton, a local engineering firm.

### THE CLOSING OF AN OUT-PATIENT DEPARTMENT AT LINCOLN.

It was reported by Mr. A. Moore, the secretary-superintendent, at the annual meeting of the governors of the Lincoln County Hospital that the board of management has found it necessary to close the out-patient department for the time being, owing to the department being taken by the War Office. Arrangements have been made, however, for any serious case from Lincoln or anywhere else to be treated at the casualty department. In practice, this means that no patient will be refused treatment; minor cases will be dealt with as speedily as possible in the circumstances. As the position is not thought to be quite clear to the public, Mr. Moore has undertaken to explain it more definitely in the newspapers. It was reported that, although the year's income was £300 more than in 1916, the expenditure—partly owing to an increase of 260 in the number of patients and partly to the greater cost of provisions, drugs, etc.—showed a considerable increase; the balance in hand of £278 was thus turned into a deficit balance of £220 by the end of the year. The board of management was authorised to arrange with the various authorities concerned a scheme for the treatment of venereal diseases at the hospital.

### LARGE GIFTS TO NEW WAR HOSPITAL.

TOWARDS the cost of the new war hospital which it has been decided to build at Sunderland—and which was the subject of a note in the preceding issue of THE HOSPITAL—about £18,000 had been received up to last week-end. Messrs. James Westoll, the shipowners, gave £3,000, Mr. Hiram Craven £2,500, Mr. Jenneson Taylor £2,000, and the following £1,000 each: The Mayor of Sunderland, Mr. R. A. Bartram, Mr. and Mrs. H. P. Everett, Mr. J. W. Witherington, Messrs. Robert Thompson and Sons, Messrs. John Blumer and Co., Sunderland Gas Company, and an anonymous donor. Obviously there will be little or no difficulty in obtaining the full £30,000 which the hospital will cost. Messrs. W. and T. R. Milburn have been appointed honorary architects, and it is announced that it has been decided to approach the Sunderland Board of Guardians with a view to utilising their hospital building. Colonel Littlewood, at a recent meeting, when a site in Ryhope Road offered by Mr. T. Backhouse was under discussion, remarked that adapted buildings were rarely as suitable, as temporary structures, designed for hospital purposes. If this plain fact had been realised, perhaps fewer schools and picturesque but unsuitable country houses would have been employed. For a private house standard is one thing, and a hospital standard is another, and very few houses were intended to be as full as their "conversion" into hospitals has made them.

### FOOD PRICES AND PATIENTS' FEES.

THE Lincoln City Council has decided to raise the charges for the treatment of naval and military patients at the City Hospital from 6s. to 7s. a day, and to ask the Guardians to pay an increased charge of 5s. per week for each patient. The reason for this decision is stated to be the high price of food. The Lindsey Insurance Committee has also been requested to increase the charge for patients at Dawber Sanatorium by 5s. a week. Side by side with this, a committee has been appointed to inquire whether land suitable for vegetable cultivation by city residents can be obtained, and, if so, to make suggestions for cultivating it. This example at Lincoln is a sign of the times, which other city councils would do well to consider.

### NEWMAN ON THE HOSPITAL IDEAL.

THE spectator is said to see most of the game because his view is detached even when, as a layer of bets, his interest inclines his hopes to one side or the other. It is therefore interesting to see what a great theologian, who carried the head of a sceptic under the hat of a cardinal, said of that hospital ideal, which has been so persistently preached in our columns, and belief in which has always lain at the root of our arguments in support of the voluntary system. In his "Idea of a University," a book which is become topical to-day in view of the educational controversy, Cardinal Newman declared that a University, even if it made the pursuit of knowledge for its own sake its chief object, might be "sanctified to the service of religion, as an hospital or an almshouse" might equally be charges pursuing "an ephemeral end." For, comes the fine conclusion, "we perfect our nature . . . by adding to it what is more than nature, and directing it towards aims better than its own." This, which is as true of knowledge as of nature, is the prime argument for the voluntary hospital ideal. We quote this at a time when the hospital ideal of the voluntary system must be inevitably contrasted with that at the root of the State hospital system of Germany. There, as our pre-war articles showed, the German hospitals are conducted on the principle that the pursuit of knowledge is its own justification, whereby every patient becomes or may become a mere case; while in England under the voluntary system the ideal at least has been, first, the welfare of each patient, and only secondly, the pursuit of knowledge. Is it not, then, worth while to pause before our educational system which, in the older Universities at least, aims at "perfecting a man whatever his task," rather than perfecting him in his particular studies, is exchanged for that orgy of specialism which inevitably degrades culture into *Kultur*, and the humanities to commercialised technical education?

### FEBRUARY 14, 1917.

THE arrangements for the third Red Cross Sale of Works of Art have to be complete by the above date. The committee in charge of them have wisely issued a list of suggestions for suitable gifts, emphasising the fact that as a practical guide the smaller the size of the objects given the greater is likely to be their potential value, not only in price, but in saving space in the showrooms. Intensive rather than extensive values are most appropriate: Greek gems, miniatures, cameos, enamels, ceramics, small bronzes, mezzotints, shawls, and the like. Once more Mr. John Lavery and Mr. Harrington Mann have offered, with Mr. Laszlo, to paint portraits of "subjects" chosen by the highest bidders.

### EXPANDING COLLECTIONS FOR VOLUNTARY HOSPITALS.

FINANCIAL records are being set up in all parts of the country in connection with the public support of hospitals, whose appeals were never more generously responded to than at this moment. A gratifying feature of this spirited recognition of the splendid work which such institutions are doing is the part played in it by the working people, who in almost every big centre are showing a much greater interest in hospitals than formerly. Four cases in point come under notice this week. At Grimsby, as recorded in detail above, several sources of income overstepped all previous bounds. The Saturday collections for the Lincoln County Hospital totalled £877 this year, an increase of £140; the amount raised in this way in Lincoln itself (viz. £500) was also a record. The third example is from Shrewsbury, where, at the 169th anniversary service of the Salop Royal Infirmary held at St. Chad's Church last week, the collection realised no less than £800. This is a magnificent record, the highest amount taken previously was £650. Lastly, the Cleckheaton District Committee raised last year for Bradford hospitals the record sum of £436. Facts like these should give heart to the managing bodies of hospitals who are, in these latter times, facing heavy additional expenditure in almost every direction. Their aim should be to keep these sources of revenue at the present high level when the normal times return, an accomplishment which should not be outside the limits of possibility given good organisation and tactful procedure.

### CAUTION IN CORNWALL.

The Penzance Guardians do not believe in doing things in a hurry. For many years they have had under discussion the proposed appointment of a permanent assistant night nurse at the workhouse infirmary, and on each occasion when the subject has come under discussion it has been successfully shelved. Last week, however, it was revived again on a recommendation by the house committee that an appointment should be made. It was stated that a permanent night nurse would be cheaper than calling in a special nurse for night duty when circumstances made that course imperative, and that there were in the infirmary or imbecile wards old people—one 107 years of age—who really ought to be looked after during night-time. The necessity for economy was urged by members of the Board, who were against any addition to the existing nursing staff of three, and an amendment was eventually passed by 21 votes to 15 again deferring the question until information has been obtained from other Cornish authorities as to the number of inmates in their institutions in proportion to the nursing staff. The Penzance Board does not intend to be rushed into premature action!

### THE BARNSTAPLE SMALLPOX HOSPITAL SHIP.

The Barnstaple Port Sanitary Authority and the Local Government Board do not see eye to eye, and the differences were brought out in a strong light at a meeting of the authority last week, which was attended by Dr. Fletcher, the Local Government Board inspector. The old hospital ship is the bone of contention, and Dr. Fletcher, who had visited it, said he found certain bad leakages in the vessel. There were times when the ship was difficult to get at, and he thought it would be better to have a permanent hospital on land. The cheapest and most satisfactory way, perhaps, would be for the authority to link up with other areas in the district and have one hospital between them. This official suggestion, however, was not well received. Lieut.-Commander Newcome retorted that they had had the ship for twenty years, and she had served them well. This "stick-to-the-old-ship" attitude was backed up by another member, Mr. P. H. Harris, who said the vessel was in as good condition now as she was twenty years ago. The great expense of a new hospital at the present time was also quoted as an obstacle. There were other similar expressions of opinion on the part of the members, and the next step, if any, will presumably have to be taken by Lord Rhondda's department.

### THE SEAMEN'S HOSPITAL.

The Seamen's Hospital is one of the latest hospitals whose secretarial staff has been drawn upon for the purposes of the war. Last week the appointment was announced of Mr. A. P. Hughes Gibb, its assistant secretary, to be private secretary to Lord Devonport, the Food Controller. It is understood that Mr. Hughes Gibb will return to the Seamen's Hospital after the war, and meanwhile he keeps in close touch with Mr. Michelli, attends one day a week at the hospital, and is continuing the work connected with the Special Appeal organised by Lord Devonport, the Chairman of the Special Appeal Committee. The office of the Food Controller is at Grosvenor House, the residence of the Duke of Westminster. During Mr. Hughes Gibb's absence from the Seamen's Hospital his work has been undertaken by Mr. Fletcher-Toomer, a member of the Institute of Civil Engineers, who has retired from the practice of his profession and has kindly placed his services at the disposal of the Seamen's Hospital. These arrangements appear to be excellent, as the administrative side of the Seamen's Hospital, Greenwich, undoubtedly is.

### THE RAILWAY FARES OF HOSPITAL WORKERS.

A hospital is always a centre of activities and possesses a large number of non-resident workers. This is doubly true nowadays when practically every institution is the nucleus of a cell of depôts, Linen Leagues, workshops, and supply centres around the periphery of its circle. The recent rise in railway fares presents a problem to some of these institutions, not only to those which draw their workers from the suburbs, but to those situated in the provinces as well. To give time is possible to almost all of us; to give money is possible to many; but to give both time and money is the opportunity of very few. Whether this class of worker can obviate the new expense which his or her patriotism is costing by taking out a season ticket is often doubtful. Is it too much to hope that a rebate be granted to those who prove their journeys will be undertaken purely for hospital purposes?

### THE CALL FROM THE COLOURS.

We hardly know which has proved the more inspiring reading: the rise of some £7,500 over previous records in the receipts of the Hospital Saturday Fund between January 11 and December 9, 1916, or the part which women have taken in achieving it. It is largely the women who have replaced the former honorary collectors. It is, therefore, under the lead of those unable to go, to the women, we should acknowledge the nation's debt for services which have made war as inspiring as peace to the public, and the continued support of the voluntary hospitals secure. The time may come with peace when the nation should remember their services, and secure, as a duty to our women, that careful redistribution of labour which is a problem so urgent as to make, in the absence of foresight and responsibility, peace itself a terrible experience, and the disbanding of the present huge Army an even more heart-searching question than its raising and maintenance have been. The women who filled the gaps in the ranks of our workers must not in their turn be unprovided for when the call *from* the Colours has to be made.

### THE ROYAL EARLSWOOD INSTITUTION.

THE board of management announce that they have adopted a scheme which revises the administration of the charitable basis of the Society by modifying the method of election. Instead of admitting a proportion of the candidates who obtain a majority of votes as the result of a poll, admission shall be secured by obtaining a definite number of votes regulated by the contribution towards cost. Therefore the candidate who secures 500 votes for which a sum of 40 guineas per annum is paid will be in as good a position as the one who has collected 1,000 votes which represent only 15 guineas. The result, presumably, will be that the honorary life governor will not be so much sought after as the governor who had paid hard cash. The voting system, with its many evils, dies hard; but this particular variation, which has, no doubt, been adopted because of its financial possibilities, will not lessen the troubles of unfortunate people who have to spend their money and time, over a period of at least eighteen months, in soliciting the support of the subscribers to the Royal Earlswood Institution for Mental Defectives. We can imagine a widow, for instance, with very little business knowledge, poring over the list of voters, and calculating in which direction her badly spared postage stamps may be best expended, and by the worried effort reducing herself to a mental condition not enormously better than that of her unfortunate offspring.

### A PATIENTS' BOOK.

MR. W. BROWNFIELD CRAIG, Assistant County Director, Totnes Division, has edited a collection of letters, sketches, verses and so on, sent by soldier patients to express their gratitude for the treatment given to them in the five V.A. hospitals of the division. Not only is he the editor, but he is also the publisher of the collection, which forms a curious chapter in hospital history. The frontispiece is a reproduction in colours of Lance-Corporal C. Milne's water-colour drawing, "Near Ypres," and illustrations are given of the hospitals, together with the patients' sketches already alluded to. The collection, which is entitled "Thank you, Sister," was happily conceived and shows how internal resources and events can be used better than anything else for hospital souvenirs and publications generally. Mr. Craig is giving all the profits from the sale of this brochure to the V.A. Hospitals Fund, Totnes.

### GARDENING UNDER MEDICAL SUPERVISION.

WE have drawn attention before now to the therapeutic value of some useful activity in convalescence, where this is properly controlled, and therefore were glad to see the plea advanced by military hospital physicians in the *Times* for gardening under medical supervision for convalescent cases among the wounded. He added the suggestion that if such men were employed in growing their own food in their hospital grounds, which are suitable in the case of many military hospitals, the national food supply would be increased. What is wanted is initiative on the part of the military medical superintendents and medical commanding officers. We wish we could be sure that it would be forthcoming, but the military mind is rather slow to undertake new departures, however promising, if they are not imposed by superior authority as part of the prescribed routine.

### CABBAGES AND POTATOES ON A HOSPITAL SITE.

A LETTER from the Local Government Board, asking for the plans and estimates of the proposed Llandough Hospital, in order that the work might be advanced without unnecessary loss of time, was referred to the building committee by the Cardiff Board of Guardians last week. The clerk to the board, however, said he doubted whether it would be possible to submit any estimates, as it could not be ascertained in the present circumstances what the cost exactly would be. The hospital approximately would involve an expenditure of £70,000. Meanwhile, the board decided to utilise the site for the cultivation of foodstuffs under the direction of one of their officers.

### "HAVE YOU SEEN OUR VEGETABLE BEDS?"

ONCE again we put forward a plea for the more general adoption of hospital gardening, with especial regard to the growing of vegetables. To give point to the suggestion, and excite the smile which will make it stick in the hearer's ear, we suggest the substitution of vegetables in every flower-bed. For while many hospitals are without gardens, almost every hospital even in London possesses a patch of grass and a bed or two of flowers. Such patches are not regarded as gardens; but we venture to suggest that they would soon be accepted as such if passers-by saw cabbages and potatoes instead of geraniums growing in them. It would be a simple, but practical, way of helping to solve the economic problem. The home-grown potato has a new dignity in war, and should be seen with cabbages and cauliflowers in every hospital which possesses a flower-bed. For this purpose every hospital with a flower-bed should be deemed to have a garden, and every bed should boast its vegetables this year.

### POTATO SUBSTITUTES IN HOSPITAL DIET.

POTATOES are becoming a sore subject, if the expression may be used in regard to a comparatively harmless vegetable. Some scarcity is admitted, and therefore it may be interesting to inquire what shifts are being employed by hospital managers who are experiencing a shortage in their supply. Rice, in itself, is an agreeable substitute, but rice is fivepence a pound. Split peas and beans are now, in the southern counties at least, equally expensive; macaroni, which in bulk is not regarded by cooks as of equal substance with rice, is even dearer. What, then, is to be done? Some hospitals have adopted the practice of substituting rice or split peas on one day a week, a practice which helps to eke out the potatoes, though it is no actual saving in cost. If better substitutes have been found practicable, what are they? We pause for a reply.

### "RECUPERATIVE HOSTELS."

A SCHEME for which much can be said in the way of commendation has lately been launched which has as its object a two-fold purpose, viz. the production of food and the cure of those sailors and soldiers who are suffering from nerve shock, etc. The summarised idea of the movement is the establishing of a number of "Recuperative Hostels" in various parts of the country, where gardening, poultry-keeping, farming, etc., will be carried on by invalided Service men, and these, while greatly benefiting from this course of "treatment," will be doing valuable national work by way of home production. It is stated that Surrey will probably be the first county where one of these hostels will be set up, and that already a "Clearing House" exists at Hampstead where men will be looked after until well enough to commence work on farm or land.

### STATE DRUG DEPOT AT SYDNEY.

THE Office of the Minister of Public Health, Sydney, has issued a circular-letter to each of the hospitals of the State, which is published in the *Prince Alfred Hospital Gazette*, Sydney, stating that the Government has decided upon the establishment of a State Drug Depot in order that Government and general hospitals may derive the advantage, as regards price and purity of supply, resulting from co-operative action in contracting through one central authority for drugs, disinfectants, and surgical dressings. It is suggested that as Government contributes approximately 60 per cent. of the funds required for the upkeep of hospitals in New South Wales, the co-operation of the committees in the new scheme may be reasonably expected. It is in contemplation that tenders shall be invited simultaneously in Europe, America, and Australia, and that supplies will commence on July 1, 1917. The experienced machinery of the Stores Supply Department of the State will be utilised in contracting for supplies and in the control of the Depot and distribution.

### THE SCARCITY OF TEACHERS OF REMEDIAL EXERCISES.

LIKE other authorities, the Bradford Education Committee has experienced difficulty in obtaining suitable candidates to work in the Clinic for Remedial Exercises, and it has been considering the desirability of strengthening the existing staff dealing with physical education. The conclusions arrived at are that scholarships of £50 a year, tenable for not more than two years, should be established to encourage suitable men or women to go through a course of special training. In this connection the training institutions suggested are: Dr. Arvedsen's Institute at Stockholm; Bergman Osterberg College at Dartford; Bedford Physical Training College; Dr. Mary Coghill-Hawks' Swedish Institute and Clinique for Medical Gymnastics, etc.; and Dr. Barrie Lambert's Training College for Massage and Remedial Gymnastics. The scheme suggests that candidates for scholarships should be interviewed by the medical sub-committee, which shall make recommendations at which institution the scholarship shall be held, after taking into consideration, amongst other things, the needs of the Education Committee and the candidate's previous educational history. The scholarships will be held on condition that the Education Committee has the first claim on the holder's services for three years after training.

### MEDICAL STATUS OF CHRISTIAN SCIENTISTS.

IT is evidence of the strength of the Christian Science teaching in certain parts of the United States that in the statutes of the State of New York forbidding the practice of medicine by anyone not duly licensed, there occurs a proviso that the statute "shall not be construed to affect the practice of the religious tenets of any Church." How this exception operates is illustrated by a recent prosecution against a Christian Science healer. He kept an office with a sign on the door describing himself as a Christian Scientist. For resort to his office, where prayer was said, there was a price paid, "either as a compensation or as an honorarium." It was held that if the defendant was sincere and did not use his Church as a shield to cover a business undertaking he could not be convicted. The judgment of the Court lays stress on the proviso referred to. One of their findings is this: "It appears that it is a tenet of the Christian Science Church that prayer to God will result in complete cure of particular diseases in a prescribed individual case. Healing would seem to be not only the prominent work of the Church and its members, but the one distinctive belief around which the Church organisation is founded and maintained." The decision is in the direction of extending rather than restricting the proviso. Had a similar case occurred here, we are inclined to think that a British Court would rule that the latitude allowed by the statute should in any event not be further enlarged, more particularly as this was a question concerning public health. Further extension, if granted at all, should be in the hands of the Legislature, not the judges. And in the case referred to there was easily room for a restrictive interpretation of the statute; for what is "the practice of a religious tenet"? It might be defined as conforming one's conduct to one's belief. In that sense certainly a Christian Scientist may seek cure by prayer. It does not mean that another may hold himself out to cure someone else by praying for him.

### WARD WIT AND "FOWL PLAY."

The editor of the 3rd London General Hospital *Gazette* has shown infinite capacity in collecting amusing anecdotes and examples of native wit in his perambulations through the institution. We take from his pages the explanation offered by a ward wit on the country's recent escape from a General Election. He put it down to the high price of eggs, having been inspired to conceive this notion by the substitution of kippers for eggs as part of the hospital menu. Recent importations have brought down the price, but dwellers in the country daily hear poultry-keepers and owners of pigs complaining that with the higher price of food the production of eggs and pork does not pay. In the case of pork, we have evidence that this is an exaggeration so far as those are concerned who from the size of their establishment or the amount of their land are able to use waste products or in part grow their own "feed." It would be interesting to hear the experience of those hospitals which have started poultry since the war. Perhaps Miss Edith Holden will tell us whether the 3rd London General Hospital has made its poultry pay.

### HOSPITAL MUSIC.

The bust, by Mr. Derwent Wood, of the ex-commanding officer of the 3rd London General Hospital, Wandsworth, has already been illustrated in the *Gazette* of that institution. The current (January) issue contains a reproduction of the portrait which has been painted of him by Corporal George Coates. An officer who can be immortalised by his staff of patients is lucky indeed, and the hospital which can attract such a staff is no less happy. But the arts and the 3rd London began and have continued in company, and therefore it seems only in the fitness of things that the Rev. J. Thompson Phipps, G.C.F., should be able to announce that the hospital has now received a grant of £320 to the organ fund on condition that the total sum raised, apparently £580, be spent on the organ alone. The grantors are the Carnegie United Kingdom Trust, and it so happens that we have lately had experience of the invaluable work being done by their allied body, the Carnegie Hero Fund Trustees, and so we are delighted to note the good sense which has dictated the condition attached to their contribution. Hospital music, like hospital architecture and every detail of its institutional equipment, should be of the highest standard. The grant of an organ of the best type is no more superfluous, according to the hospital standard, than the gift of the best theatre table or patient's locker.

### THE IMPORTANCE OF A STANDARD.

We take the liberty to emphasise the value of an organ of first-rate quality, as a part of hospital equipment, since the smaller-minded hospital officer is apt to suppose that "money could be spent more usefully" on something else, a new boiler, for example. While we admit that the foundations of a building must be placed in position before the roof, we deny that one is more necessary than the other, and the same principle applies to hospital music and hospital chapels, memorial tablets, and the like. A thoughtful superintendent once remarked to us on the evil effects on his staff which had been produced by holding services in a committee room. He said that religious devotion was an integral part of hospital life, and that to have no special chapel devoted entirely to religious use was hardly worse than having no operating theatre. The younger men can with difficulty realise how commonly a room, normally used for other purposes, was adapted to surgical operations a few years ago. They take the theatre as a matter of course. In some smaller hospitals or infirmaries a room is still employed "when necessary," though this is happily a moribund custom. The hospital chapel, however, is still regarded as something of a luxury. We therefore put in a plea for as high a standard of effort in regard to hospital music as is now accepted as necessary in surgical technique or hospital account-keeping. Where there's a will there's a way; and the Carnegie United Kingdom Trust deserve our thanks for encouraging hospital music by the generous grant towards a good organ in the 3rd London General Hospital at Wandsworth.

### "WHAT MAKES AN ARMY."

The war hospital gazettes for the most part reflect little beyond the humour and the literary aspirations of those who write the articles. In the sketches and caricatures, since the gift required to produce them is not taught, like mere composition, in childhood, there is a higher standard as a rule. Sometimes, however, as it were an aside, a genuine piece of criticism, which illumines larger issues, is slipped into an article. As an instance we take the following remark made in the first article of the *Southern Cross* for December. The writer, who gives his impressions on arriving at the 1st Southern General Hospital, Birmingham, says that, in contrast to the machine-made minds which military red-tape is apt to produce at home, "the returned Expeditionary Force officers, having felt the grip of war and danger, and having seen the spirit of sacrifice, . . . their souls rebound with human thought and kindness: a spirit which alone makes, and will always make, our Army." Military discipline is accepted so universally as necessary to an army in the field that in this connection it is interesting to quote the criticism of those who condemn, or have condemned it in peace-time, preferring the contrast offered by what is somewhat loosely called a citizen army. It would seem from the writer quoted that military discipline, which he notes as free from most of its defects as a system in the field, compares favourably there with military discipline at home, that is under peace conditions. Voluntary hospitals with the voluntary discipline of institutional life will appreciate the distinction, and the striking praise implied to their war discipline in the above.

